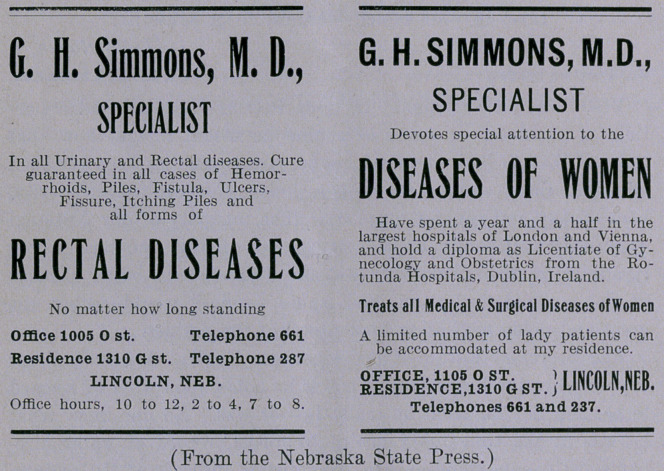# The Lifting of the Veil

**Published:** 1909-12

**Authors:** 


### THE LIFTING OF THE VEIL.

"Ye would be dupes and victims and ye are.
Is it enough? Or must I, while a thrill
Lives in your sapient bosoms, cheat you still?"
—*Lalla Rookh.*

**Our Peerless Leader.** **A Modern Mokanna.**

Yes, it is quite enough. If "Our Peerless Leader" had a particle of manhood or sense of shame he would resign, now that the veil that so long obscured his beautiful past has been stripped off, and there stands revealed a hideous record of hypocrisy, homeopathy and rankest quackery. The integrity of our National organization depends upon getting rid of this incubus, whose tyrannical methods have outraged and disgusted the better element of the medical profession, and whose "ethics" would reduce the great and noble profession to the level of the pathies.

The Secretary-Editor of the American Medical Association was appointed to the high place of power he holds upon what he seemed to be, and not for what he really is,—or was. No one knew what he was at the time his Lincoln, Nebraska, record of water-cure-homeopathy-compound-oxygen specialist in diseases of the rectum, specialist in diseases of women, guaranteeing "a cure in every case, no matter how long standing," was advertised in the newspapers after the manner of the charletan and quack. Had this been known, does any one suppose for a moment that he would have been appointed to succeed the great Hamilton and the greater Davis? The veil of twenty years—ten or more of pretended "regularity"—which obscured the hideous past has been ruthlessly stripped off, as was that of pretended holiness of the False Prophet of Khorassan, Mokanna, who had so long deluded his fanatical followers, and he stands revealed in a record of all that legitimate practitioners of rational medicine hold in abhorrence,—as hideous as was the face of Mokanna,—so shocking that Zelica, his "Bride of Heaven," swooned on beholding it.

In addition to the Lincoln Medical Institute and water-cure-compound-oxygen advertisement in the Nebraska papers, which I reproduced in the January "Red Back," the "Peerless Leader" was running the two ads herewith reproduced. These are the latest findings from the graveyard of dead quackery, unearthed at Lincoln, Nebraska. Read them: "A cure guaranteed in every case no matter how long standing," and "Have spent a year and a half in the largest hospitals of London and Vienna," and "Hold a diploma as Licentiate of Gynecology and Obstetrics from the Rotunda Hospitals of Dublin, Ireland." "A limited number of lady patients can be accommodated at my residence." Shades of Briggs, go blush!

---

**G. H. Simmons, M. D.,**

**SPECIALIST**

In all Urinary and Rectal diseases. Cure guaranteed in all cases of Hemorrhoids, Piles, Fistula, Ulcers, Fissure, Itching Piles and all forms of

**RECTAL DISEASES**

No matter how long standing

Professor G. Frank Lydston, of the Illinois Medical College, has issued several pamphlets in which he asserts and presents documentary evidence to prove that Dr. Simmons "matriculated by proxy at Rush Medical College, Chicago," in the fall of 1891, and received a diploma from that school in March, 1892, a large part of which time he was practicing homeopathy in Lincoln, Nebraska, more than three hundred miles away. This Dr. Lydston substantiates by photographic reproductions of death certificates, with dates, and prescriptions with dates ("diphtheria" spelled "diptheria," and one prescription for "Firwein," whatever that is), and all the record Dr. Lydston could find of "Our Peerless Leader's" stay in Chicago (there was none at Rush) covered a period of twenty-two days—ten one time and twelve at another. Well may Dr. L. ask, "Was Rush a diploma mill in 1891-92?" Charges based upon this evidence have been brought against Dr. Simmons in the Chicago Medical Society and in the Illinois State Medical Association.

(A copy of Lydston's pamphlets can be had for the asking.)

"And this is the man who is the head and front of American medical journalism; who dictates our policies, controls medico-political appointments, supervises our organization, censors our articles, handles the business of our great journal, tells us what there is of value in our armamentarium therapeuticum, tells us what shall be advertised and what shall not, supervises the ethics and morals of our drug manufacturers, tells our independent medical journals what they shall advertise and what they shall not; receives invitations to lecture before our medical societies on the proper methods of teaching therapeutics—in short, who is the arbiter of all things literary, ethical, political, therapeutic and moral in American medicine.—*Lydston.*

And this is the man and this is the conduct that is defended by the controlled press,—the collar editors like Chase, and the Kentucky "Mac" who edits the Kentucky Tentacle—a brother of "Pardoned" Mac-the-Mick, chief spieler and walking delegate; and the Indiana personage, one Bullar, I think his name is, who runs the Hoosier State Tentacle. Will the reputable element of American medicine stand for it?—*Texas Medical Journal,* May, 1909.

## Figures and Tables

**Figure f1:**